# *Trypanosoma cruzi *alkaline 2-DE: Optimization and application to comparative proteome analysis of flagellate life stages

**DOI:** 10.1186/1477-5956-6-24

**Published:** 2008-09-08

**Authors:** Adriana D Magalhães, Sébastien Charneau, Jaime Paba, Rafael AP Guércio, Antonio RL Teixeira, Jaime M Santana, Marcelo V Sousa, Carlos AO Ricart

**Affiliations:** 1 Laboratory of Biochemistry and Protein Chemistry, Department of Cell Biology, University of Brasília, 70910-900, Brazil; 2 Chagas Disease Multidisciplinary Research Laboratory, Faculty of Medicine, University of Brasília, 70910-900, Brazil; 3 Laboratory of Parasite-host Interaction, Department of Cell Biology and Faculty of Medicine, University of Brasília, 70910-900, Brazil; 4 Department of Biochemistry, Federal University of Paraná, Curitiba, Brazil

## Abstract

**Background:**

*Trypanosoma cruzi*, a flagellate protozoan, is the etiological agent of Chagas disease, a chronic illness that causes irreversible damage to heart and digestive tract in humans. Previous 2-DE analyses of *T. cruzi *proteome have not focused on basic proteins, possibly because of inherent difficulties for optimizing 2-DE in the alkaline pH range. However, *T. cruzi *wide pH range 2-DE gels have shown few visible spots in the alkaline region, indicating that the parasite either did not have an appreciable amount of alkaline proteins or that these proteins were underrepresented in the 2-DE gels.

**Results:**

Different IEF conditions using 6–11 pH gradient strips were tested for separation of *T. cruzi *alkaline proteins. The optimized methodology described here was performed using anodic "paper bridge" sample loading supplemented by increased concentration of DTT and Triton X-100 on Multiphor II (GE Healthcare) equipment and an electrode pad embedded in DTT- containing solution near the cathode in order to avoid depletion of reducing agent during IEF. Landmark proteins were identified by peptide mass fingerprinting allowing the production of an epimastigote 2-DE map. Most identified proteins corresponded to metabolic enzymes, especially those related to amino acid metabolism. The optimized 2-DE protocol was applied in combination with the "two-in-one gel" method to verify the relative expression of the identified proteins between samples from epimastigote and trypomastigote life stages.

**Conclusion:**

High resolution 2-DE gels of *T. cruzi *life forms were achieved using the optimized methodology and a partial epimastigote alkaline 2-DE map was built. Among 700 protein spots detected, 422 were alkaline with a p*I *above 7.0. The "two-in-one gel" method simplified the comparative analysis between *T. cruzi *life stages since it minimized variations in spot migration and silver-stained spot volumes. The comparative data were in agreement with biological traits of *T. cruzi *life forms and also corroborated previous *T. cruzi *proteomic studies. For instance, enzymes related to amino acid metabolism and dehydrogenases were more abundant in epimastigote 2-DE gel whilst *trans*-sialidase and a paraflagellar protein were found specifically in the trypomastigote 2-DE profile.

## Background

*Trypanosoma cruzi*, a flagellate kinetoplastid protozoan, is the etiological agent of Chagas disease, a chronic illness that causes irreversible damage to heart and digestive tract in humans. Chagas disease remains as a serious problem in Latin America where approximately 17 million people are infected [1]. The drugs currently used for its chemoprophylactic treatment are highly toxic and present variable efficacy [2] whilst vaccines against the parasite are not available yet.

*T. cruzi *life cycle occurs inside both bloodsucking triatomine insect vectors and mammalian hosts. During its life cycle, the parasite differentiates into four stages: epimastigote and metacyclic trypomastigote in the insect vector, and bloodstream trypomastigote and amastigote inside the mammalian host. It has been assumed that the biological features of each trypanosome developmental stage were shaped by changes in protein expression during its life cycle. These features have led to proteomic analysis of the parasite using different strategies such as two-dimensional gel electrophoresis coupled to mass spectrometry (2-DE-MS) [3,4] and isotope coded affinity tagging (ICAT) [5] even before the *T. cruzi *genome sequence was published [6].

It appears that proteomic approaches have advantages over nucleic acid based strategies for the study of differential gene expression in trypanosomatides since their gene regulation is mostly post-transcriptional, through control of mRNA stability and translation efficiency. Consequently, there is not a good correlation between mRNA and protein levels [7,8].

More recently, the Multidimensional Protein Identification Technology (MUDPIT) was used to identify 2784 proteins from the four *T. cruzi *life forms. Although the *T. cruzi *genome sequence was available, several proteins were identified by cross-species homology and remain putative [9]. It was previously shown that the number of proteins identified by shotgun protein sequencing methods is higher than that usually achieved by 2-DE-MS based proteomics [10]. On the other hand, the use of 2-DE-MS approach appears to bear important advantages over high throughput proteomic strategies (such as MUDPIT) that hydrolyze the proteins into peptides before chromatographic separation and identification. Since 2-DE permits the separation and the visualization of intact polypeptides, it handles better situations dealing with quantitative and qualitative changes of modified proteins during the biological process time course [10] as reported for amastigogenesis [3] and metacyclogenesis [11] differentiation processes in *T. cruzi*.

Obviously, the collection of proteomic techniques presently available should be used in complementary way. Finally, it must be said that the *T. cruzi *proteome characterization is far from complete considering that the proteome is a very dynamic entity that will change in response to organism life stage or breed and environmental conditions.

So far, reports on 2-DE analysis of *T. cruzi *proteome have not focused on basic polypeptides, possibly because of inherent difficulties for optimizing 2-DE conditions in the alkaline pH range. Oddly, previous reports have shown that *T. cruzi *wide range pH (3–10) gradient gels displayed few spots in the alkaline region [3,4] indicating that the parasite either did not have an appreciable amount of alkaline proteins or that these proteins were underrepresented in the 2-DE gels.

In the present article we show an optimized and reproducible protocol for the 2-DE of *T. cruzi *proteins in the 6–11 pH range. Landmark proteins were identified by peptide mass fingerprinting allowing the production of an epimastigote 2-DE map. Additionally, the "two-in-one gel" technique [12] was applied to determine the relative expression of the identified proteins between epimastigote and trypomastigote life forms

## Results and discussion

### Optimization of *T. cruzi *alkaline 2-DE

*T. cruzi *proteome maps in the restricted alkaline pH range have not been reported yet. However, previous attempts to separate *T. cruzi *proteins using wide pH range (3–10) 2-DE gels produced profiles with few visible spots in the alkaline region besides severe horizontal streaking problems [3,4]. Those wide range 2-DE profiles indicated that the concentration of trypanosome basic proteins was lower than the acidic ones. However, it is very likely that the alkaline polypeptides comprise a group at least as numerous as the acidic set. It has been previously shown for several organisms that the theoretical distribution of protein p*I *in whole proteomes is usually bimodal with very low fractions of proteins close to pH 7.4 [13,14]. Virtual 2-DE gels (plot of the calculated p*I *values against the calculated molecular masses of the proteins) were built for proteomes of the protozoan parasites *Leishmania major *and *T. brucei*, that belong to the same family (Trypanosomatidae) and order (Kinetoplastidae) as *T. cruzi *and share similarities concerning subcellular and genome architecture [15]. Such virtual 2-DE gels displayed a relatively symmetric bimodal p*I *distribution between basic and acidic polypeptides [16]. Thus, it would be expected that similar virtual 2-DE spot distribution would occur with *T. cruzi*.

In the present work, we aimed at obtaining a *T. cruzi *high resolution alkaline 2-DE map. Difficulties in getting suitable alkaline 2-DE maps are due to a combination of factors including depletion of charged reducing agents such as DTT in the high pH range of the IPG strip and reverse electroendosmotic flow that occurs during IEF [17]. In order to minimize these effects, we tested different experimental conditions in the 6–11 pH range.

*T. cruzi *epimastigote life stage was used for optimization due to the relative facility to culture it in large scale. Using this parasite stage, we have observed that the intensity of spots in the alkaline region was visually lower than in the acid pH range. Therefore, we introduced the first relevant protocol modification by increasing the protein loading in the 6–11 IPG strip to twice the amount previously loaded in 4–7 pH range 2-DE gels [3]. We tested several IEF methods using epimastigote protein extracts and the most representative of them are detailed in Table 1.

**Table 1:** Methods tested for alkaline IEF of *T. cruzi *proteins

Method	Sample application^a^	Strip rehydratation	Equipment	Cathodic DTT pad	IEF program		
					
					Voltage (V)	Time (h:min)	Volt-hours (Vh)
A	In-gel rehydration sample in 350 μL of SB1	12 h	IPGphor	no	50 μA/strip, all step-n-hold		
					500	1:00	500
					1,000	1:00	1,000
					8,000	4:00	32,000
B	In-gel rehydration sample in 350 μL of SB1	12 h	Multiphor II	65 mM DTT	2 mA and 5 W all step-n-hold		
					500	0:01	8
					3,500	7:30	26,250
C	Anodic cup-loading sample in 100 μL of SB1	12 h in SB1 without sample	Multiphor II	65 mM DTT	2 mA and 5 W all gradient		
					300	3:00	450
					1,400	16:00	11,200
					3,500	5:00	8,750
D	Anodic cup-loading sample in 100 μL of SB2	12 h in SB2 without sample	Multiphor II	SB2	2 mA and 5 W all gradient		
					300	3:00	450
					1,400	16:00	11,200
					3,500	5:00	8,750
E	Paper-bridge loading sample in 250 μL of SB1	10 h in SB1 without sample	Multiphor II	SB1	2 mA and 5 W all step-n-hold		
					150	1:00	150
					300	2:00	600
					600	1:00	600
					3,500	10:30	36,750
F	Paper-bridge loading sample in 250 μL of SB3	10 h in SB1 without sample	Multiphor II	SB1	2 mA and 5 W all step-n-hold		
					150	1:00	150
					300	2:00	600
					600	1:00	600
					3,500	10:30	36,750

^a ^500 μg protein was applied in 6–11 linear IPG strips, 18 cm, at 20°C

SB1 = 2-DE sample buffer 1 (7 M urea, 2 M thiourea, 0.5% IPG buffer 6–11, 65 mM DTT, 2% Triton X-100, 10% isopropanol and bromophenol blue)

SB2 = 2-DE sample buffer 2 (7 M urea, 2 M thiourea, 0.5% IPG buffer 6–11, 1.2% DeStreak, 2% Triton X-100 and bromophenol blue)

SB3 = 2-DE sample buffer 3 (7 M urea, 2 M thiourea, 0.5% IPG buffer 6–11, 85 mM DTT, 2.5% Triton X-100, 10% isopropanol and bromophenol blue)

The assessed methods differed according to the type of IEF equipment, sample buffer composition, sample application method, voltage conditions and prevention of DTT depletion. In all protocols isopropanol (10% v/v) was used in the sample buffer to minimize electroendosmotic flow as previously described [18].

Method A made use of the IPGphor equipment (GE Healthcare) and followed the manufacturer's instructions for 6–11 pH range. IPGphor has advantages over Multiphor II (GE Healthcare), another equipment routinely used in our laboratory, since it permits a single application step for in-gel rehydration/focusing and allows shorter runs due to higher voltages. Previous studies have also shown improved sample entry with the IPGphor, although there are conflicting reports about this matter in the literature [19].

**Figure 1:**
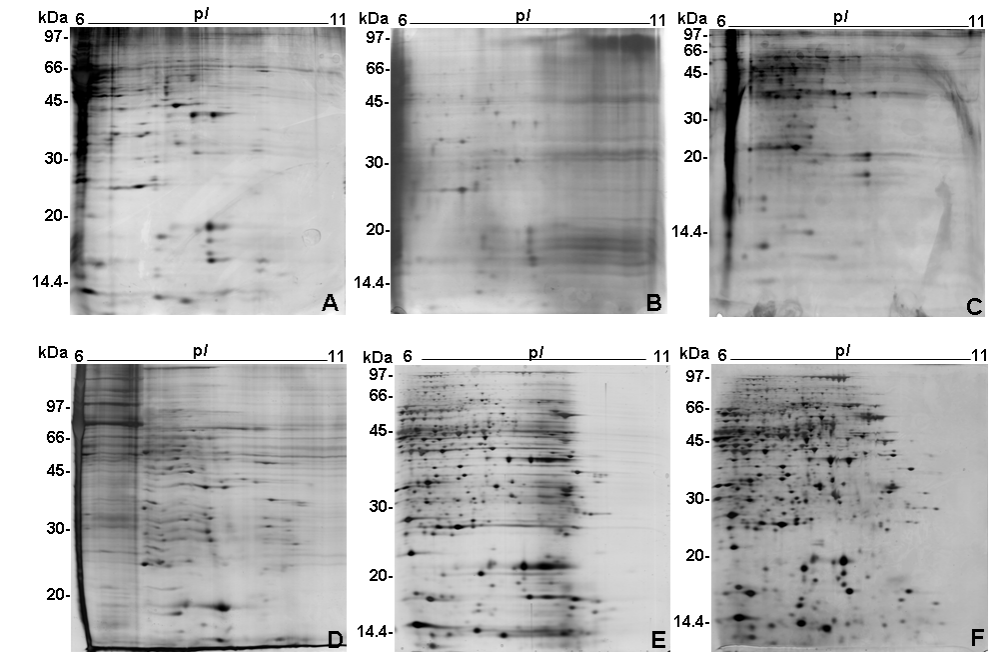
Optimization of epimastigote alkaline 2-DE profiles. The 2-DE were performed using 6–11 pH IPG strip in IEF and 12% T SDS-PAGE in the second dimension and silver stained. One representative gel is shown for each IEF method used. A, B, C, D, E and F refer to the IEF method used according to Table 1. The sample amount was 500 μg protein/gel.

Fig. 1A presents the 2-DE profile resolved by method A, showing few intense silver stained spots and mild horizontal streaking, specially in the high apparent molecular mass region.

Despite the above arguments, Multiphor II was also used to check whether the separation could be improved using a different IEF equipment. As mentioned, Multiphor II uses lower maximal voltages (3,500 V) than IPGphor (8,000 V) and consequently produces longer runs. As in method A, the sample loading in method B was done by in gel rehydration. The resulting 2-DE profile using method B presented in Fig. 1B showed a profile with much less intense spots and accentuated streaking in the more basic region.

Methods A and B used "in gel rehydration" for sample application, thus the IPG strip was directly rehydrated with a solution that contained the sample [20]. Although in gel rehydration has become the method of choice for sample application, it has been noticed that in some cases better results could be achieved by applying the sample on the surface of the IPG gel using sample cups ("cup loading") [21]. In addition, streaking problems in 2-DE gels were possibly caused by depletion of DTT in the basic region. This depletion can be prevented by the use of an electrode paper pad at the cathode as source of DTT during focusing [22]. Therefore, method C made use of sample-cup application near the anode and an electrode pad soaked in 2D sample buffer next to the cathode. In order to concentrate the sample proteins to a volume compatible to cup loading (100 μL), the sample was previously precipitated with TCA/acetone prior to IEF. Although the resulting 2-DE profile (Fig. 1C) showed that cup-loading provided higher spot resolution comparing to in gel rehydration (Fig. 1B) it was still not suitable for the production of a satisfactory proteome map.

In method D, hydroxyethyl disulfide (DeStreak, GE Healthcare) was added to sample buffer (SB2, Table 1) instead of DTT. DeStreak was previously shown to prevent streaking in alkaline regions by means of oxidation of protein thiol groups to mixed disulfides [23]. However, the presence of hydroxyethyl disulfide did not improve spot resolution in epimastigote alkaline 2-DE gels as shown in Fig. 1D.

Method E made use of "paper bridge loading" for sample application in IEF. Therefore, epimastigote proteins diluted in 250 μL of SB1 (Table 1) were applied to an arrow head paper electrode strip and positioned at the anodic end of the IPG strip as previously described [24]. Since "paper bridge loading" permits the application of higher sample volumes than cup loading, protein concentration with TCA/acetone was not necessary prior to IEF. The combination of "paper bridge loading" with higher total voltage (see Table 1) resulted in an improved 2-DE profile with approximately 480 well resolved spots, although still displaying streaking particularly in the basic end (Fig. 1E) which made difficult the detection of the most alkaline proteins.

Sample buffer composition was then modified (SB3, Table 1) by increasing DTT and Triton X-100 concentrations in order to decrease protein oxidation and precipitation respectively (method F, Table 1). The resulting 2-DE gel (Fig. 1F) displayed high spot resolution as well as a reproducible profile. 2-DE gels were run in triplicate using method F and then subjected to computational image analysis which permitted the detection of 700 silver-stained spots from 6–11 pH range with 422 alkaline silver-stained proteins over pH 7.0 and thus the majority of spots (60.2%) are in the basic range.

### Epimastigote 2-DE mapping

**Figure 2:**
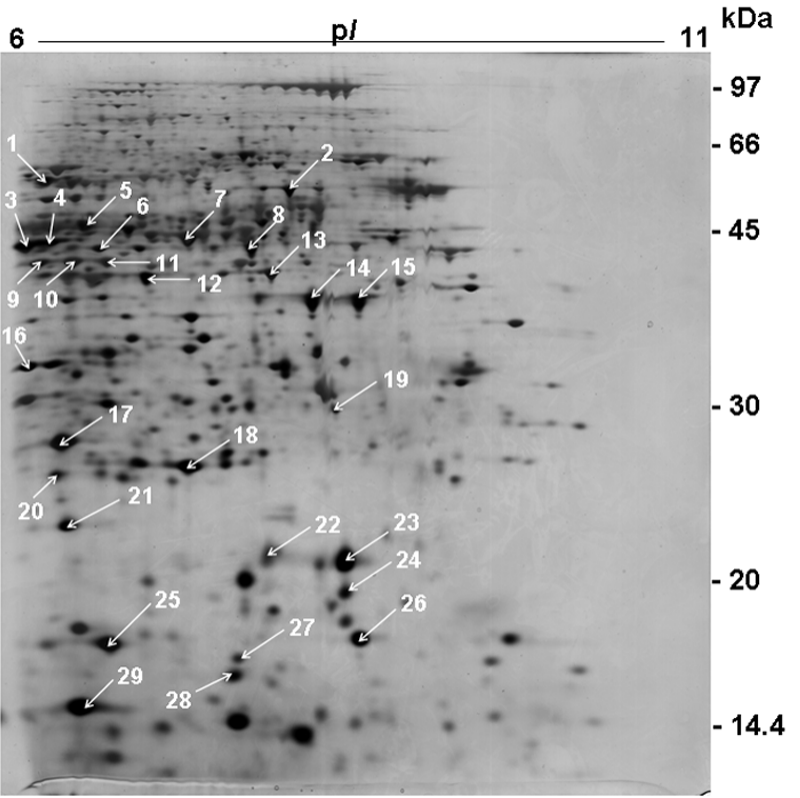
Epimastigote alkaline 2-DE map. 2-DE was carried out using method F (see Table 1). Spot numbers correspond to the proteins identified by PMF presented in Table 2.

Landmark spots were chosen (Fig. 2) and subjected to trypsin digestion followed by peptide mass fingerprinting (PMF). The identified spots are listed in Table 2. Most of them corresponded to metabolic enzymes, especially those related to amino acid metabolism. A small GTP binding protein, cyclophilin A and heat shock proteins were also detected.

**Table 2:** *T. cruzi *identified proteins and differential expression analysis among epimastigote and trypomastigote stages

**Spot^a^**	**Protein**	**Accession No. (NCBI)**	**MASCOT**		**MW**	**p*I***	**Expression**	**E/T (SEM)^h^**	**t-value^i^**	**P^j^**
							
			**Seq. cov^b ^**(%)	**Score^c^**	**Exp (Theo)^d^**	**Exp (Theo)^d^**				
**1**	*Alanine aminotransferase*	gi|71660439	16	76	55.7 (55.1)	6.2 (6.1)	E^e^	-	-	-
**2**	*Citrate synthase*	gi|71660323	13	76	53.6 (53.1)	8.0 (8.6)	E<T^g^	0.35 ± 0.09	-7.144	0.019
**3**	*Dehydrogenase*	gi|61741948	35	116	43.5 (42.4)	6.0 (6.1)	E^e^	-	-	-
**4**	*Dehydrogenase*	gi|61741944	29	81	43.9 (42.6)	6.2 (6.0)	E^e^	-	-	-
**5**	*D-isomer specific 2-hidroxyacid dehydrogenase*	gi|71420052	32	82	46.1 (38.8)	6.4 (6.0)	E>T^g^	3.81 ± 0.10	27.755	0.001
**6**	*Cystathionine beta-synthase*	gi|71425069	30	81	43.2 (42.8)	6.6 (6.4)	E<T^g^	0.24 ± 0.03	-24.786	0.002
**7**	*2-amino-3-ketobutyrate coenzyme A ligase*	gi|71650629	29	87	44.3 (44.0)	7.2 (6.7)	E = T^f^	2.58 ± 0.55	2.897	0.101
**8**	*Aspartate aminotransferase, mitochondrial*	g|71412236	22	87	43.3 (46.5)	7.7 (8.6)	E = T^f^	1.00 ± 0.21	0.015	0.989
**9**	*Arginine kinase*	gi|71407949	23	76	41.8 (40.5)	6.1 (6.3)	E = T^f^	0.84 ± 0.10	-1.544	0.262
**10**	*D-isomer specific 2-hidroxyacid dehydrogenase*	gi|71420052	47	140	42.0 (38.8)	6.4 (6.4)	E = T^f^	5.97 ± 2.45	2.027	0.180
**11**	*D-isomer specific 2-hidroxyacid dehydrogenase*	gi|71420052	42	99	41.9 (38.8)	6.6 (6.0)	E = T^f^	1.77 ± 0.39	1.962	0.189
**12**	*L-threonine 3-dehydrogenase*	gi|71406160	29	103	40.5 (37.3)	6.9 (6.7)	E>T^g^	9.64 ± 1.65	5.228	0.035
**13**	*Succinyl-CoA synthetase alpha subunit*	gi|71667777	29	81	40.9 (32.3)	7.8 (8.4)	E = T^f^	0.91 ± 0.05	-1.762	0.220
**14**	*Mitochondrial malate dehydrogenase*	gi|71414199	47	114	38.3 (31.9)	8.1 (7.6)	E = T^f^	1.27 ± 0.13	2.022	0.181
**15**	*Mitochondrial malate dehydrogenase*	gi|71414199	38	106	38.3 (31.9)	8.5 (7.6)	E = T^f^	1.43	-	-
**16**	*Pyridoxal kinase*	gi|71413338	19	76	33.1 (33.6)	6.0 (6.0)	E = T^f^	4.10 ± 1.03	2.996	0.096
**17**	*Tryparedoxin peroxidase*	gi|17224953	44	101	27.8 (22.7)	6.3 (6.0)	E = T^f^	1.60 ± 0.51	1.173	0.361
**18**	*Small GTP-binding protein*	gi|71413249	30	76	26.2 (22.0)	7.2 (7.0)	E = T^f^	1.16 ± 0.34	0.479	0.679
**19**	*Chain B, triosephosphate isomerase*	gi|4389145	26	123	30.0 (27.4)	8.3 (8.6)	E = T^f^	0.64 ± 0.10	-3.543	0.071
**20**	*Hypothetical protein*	gi|71414910	51	111	25.6 (21.5)	6.3 (6.9)	E^e^	-	-	-
**21**	*Peptide methionine sulfoxide reductase*	gi|71405176	60	110	22.7 (20.2)	6.3 (6.1)	E = T^f^	4.10 ± 1.02	3.040	0.093
**22**	*Cyclophilin A*	gi|71659715	34	85	21.1 (19.0)	7.8 (8.4)	E = T^f^	3.20 ± 1.52	1.448	0.285
**23**	*Cyclophilin A*	gi|71659715	37	82	20.8 (19.0)	8.4 (8.4)	E = T^f^	1.28 ± 0.28	0.994	0.425
**24**	*Cyclophilin A*	gi|71659715	41	80	19.3 (19.0)	8.6 (8.4)	E = T^f^	1.28 ± 0.26	1.071	0.396
**25**	*Calpain-like cystein peptidase*	gi|71407848	51	97	17.1 (15.1)	6.6 (6.4)	E^e^	-	-	-
**26**	*Nucleoside diphosphate kinase*	gi|71667532	54	94	17.3 (17.0)	8.5 (8.5)	E = T^d^	3.75 ± 2.04	1.346	0.311
**27**	*20 kDa heat shock protein*	gi|71418782	42	78	16.6 (16.0)	7.8 (7.8)	E^e^	-	-	-
**28**	*Hypothetical protein*	gi|71407758	52	104	16.0 (13.0)	7.7 (7.8)	E^e^	-	-	-
**29**	*10 kDa heat shock protein*	gi|71410853	49	78	14.8 (10.7)	6.4 (8.0)	E = T^f^	1.67 ± 0.39	1.729	0.334
**30**	*69 kDa Paraflagelar rod protein*	gi|71650963	16	80	71.0 (70.1)	6.0 (5.9)	T^e^	-	-	-
**31**	*Trans-sialidase*	gi|71409133	11	88	75.0 (81.4)	7.0 (8.4)	T^e^	-	-	-
**32**	*Hypothetical protein*	gi|71651158	47	87	13.1 (16.4)	9.6 (9.0)	T^e^	-	-	-

^a^Spot numbers correspond to those indicated in Fig. 2 and Fig. 3

^b^Percentage of predicted protein sequence covered by matched peptides via Mascot

^c^Probability Based Mowse Score of Mascot software

^d^Theoretical Mr and p*I *calculated from amino acid sequence

^e^Spots specifically detected in epimastigote (E) or trypomastigote (T)

^f^Protein expression not statistically different between both life stages by *t*-test (p < 0.05)

^g^Statistically significant (*t*-test, p < 0.05) increased spot volume in epimastigote (E>T) and trypomastigote (E<T)

^h^Epimastigote/trypomastigote (E/T) spot volume ratio with standard error of the mean (SEM)

^i^Decision value of the *t*-test

^j^Probability value that E/T ratio is different from 1 in a random event

The statistical correlation between the theoretical and experimental Mr values (Pearson r = 0.984, P < 0.001) was higher than that of p*I *values (Pearson r = 0.852, P < 0.001) (see Additional file 1). The lower correlation between theoretical and experimental p*I *values is probably due to post-translational modifications (PTMs) [25,26]. Discrepancies between theoretical and experimental p*I *values were previously observed in the trypanosomatide *Leishmania major *and were shown to be caused by PTMs, specially acetylation, N-terminal pyroglutamylation, N-terminal processing of methionine, deamidation and oxidation of tryptophan [27]. Previous *T. cruzi *proteome analyses have reported the detection of protein isoforms in 2-DE gels, probably corresponding to different post-translational modifications [3,11].

### Comparative analysis of epimastigote and trypomastigote 2-DE maps using "two-in-one gel"

Overall, we succeeded to produce a high resolution alkaline 2-DE map for the epimastigote life stage. Moreover, we applied the optimized method to other *T. cruzi *life stages resulting in well resolved and reproducible 2-DE profiles (data not shown).

The optimized alkaline 2-DE methodology was also applied to compare the relative expression of the identified proteins in samples from the insect non-invasive motile life stage, epimastigote and the mammalian invasive motile stage, trypomastigote. For that, we used the "two-in-one gel" technique described elsewhere [12]. This technique minimizes variations in spot migration and intensity when two samples are compared by 2-DE, because both samples run in the same second dimension SDS-PAGE gel and are simultaneously stained. These features are especially advantageous for the comparison of silver stained 2-DE gels. Although very sensitive, silver staining is not an endpoint procedure, *i.e*. spot intensity depends on developing time. The use of "two-in-one gel" technique minimized this drawback since both epimastigote and trypomastigote proteins were present in the same gel and were consequently subjected to the same staining conditions, including developing time.

**Figure 3:**
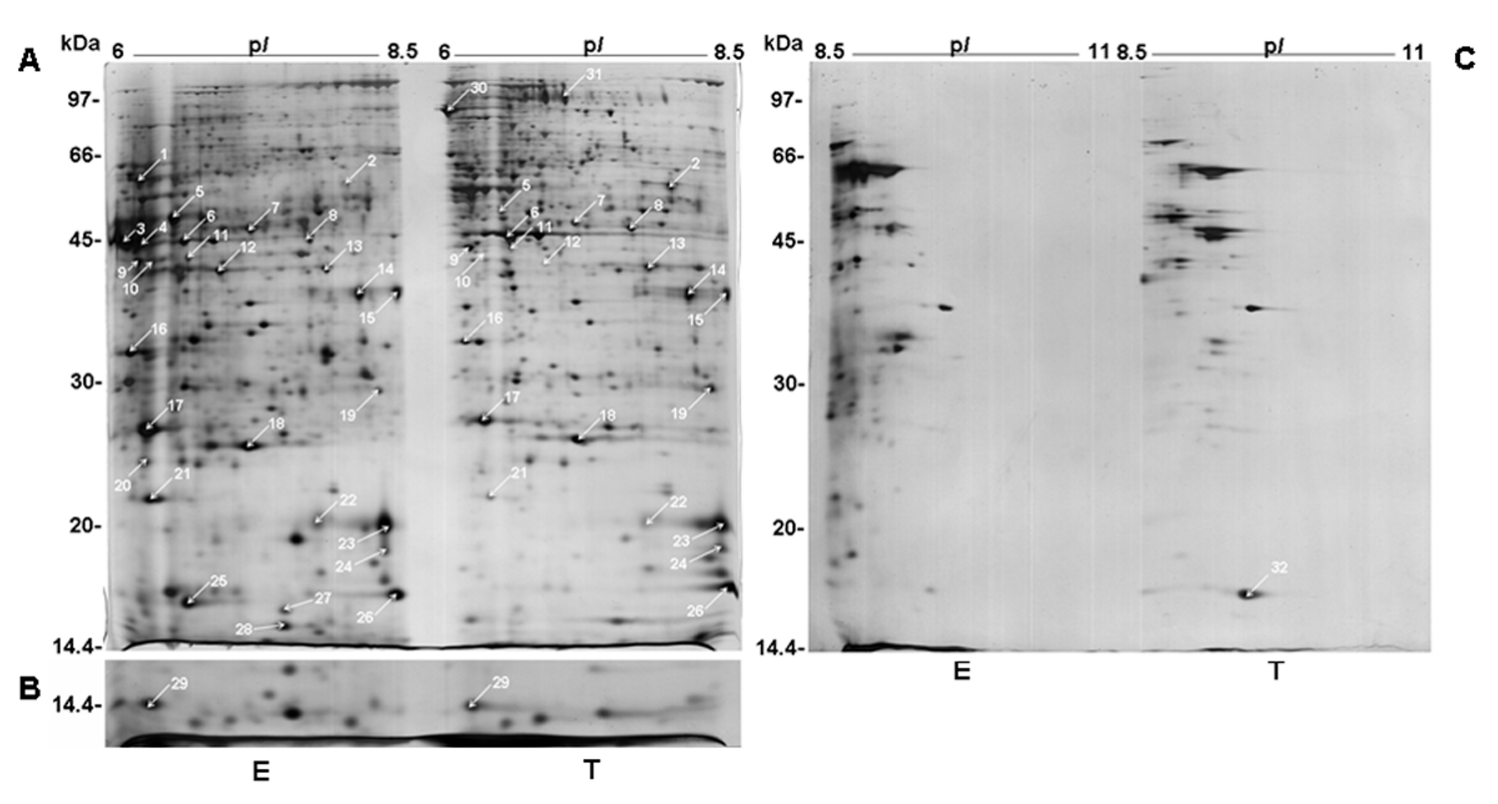
2-DE comparison between *T. cruzi *motile life forms using alkaline "two-in-one gel". IPG strips with epimastigote (E) and trypomastigote (T) samples were applied side-by-side on 12% T SDS-PAGE (see Material and Methods). A: "two-in-one gel" of 6–8.5 pH range. Spot 29 is shown in an inset (B) of a separate experiment. C: "two-in-one gel" of 8.5–11 pH range. Gels were silver stained.

Fig. 3 shows the comparative "two-in-one gel" between epimastigote and trypomastigote forms in two pH ranges. It was noticed that the majority of the spots were present in the more acidic half of the 6–11 IPG strip, corresponding to pH 6–8.5 (Fig. 3A). As expected, the "two-in-one gel" simplified the computational matching of spots between epimastigote and trypomastigote samples. Besides the spots previously identified from the epimastigote 2-DE map (Fig. 2), three additional spots detected only in trypomastigote were also identified by PMF (spots 30, 31 and 32). The relative expression of *T. cruzi *proteins was calculated using the epimastigote/trypomastigote spot volume ratio (E/T). To acquire more confidence in the E/T values, "two-in-one gel" experiments were carried out in triplicate and one-sample *t*-test was carried out to check if the E/T ratio of each spot was statistically different from 1 (Table 2). Prior to one-sample *t*-test, Shapiro-Wilk test [28] was applied to E/T ratio and confirmed that the samples came from a normally distributed population (see Additional file 2).

The results presented in Table 2 show the relative expression (differential expression or stage specificity) of the identified spots in both *T. cruzi *stages. Among 29 landmark spots identified from epimastigote 2-DE map (Fig. 2), 7 spots (1, 3, 4, 20, 25, 27 and 28) were not detected in trypomastigote gels (Fig. 3). In addition, other 4 spots displayed statistically significant differential expression between both 2-DE profiles. Thus, spots 5 and 12 were more intense in epimastigote and spots 2 and 6 in trypomastigote. Moreover, 3 trypomastigote-specific proteins (spots 30, 31 and 32) were identified as mentioned above.

The Krebs cycle enzyme citrate synthase (spot 2) showed higher expression in trypomastigote than in epimastigote gels. On the other hand, enzymes from amino acid metabolism such as alanine aminotransferase (spot 1) and L-threonine 3-dehydrogenase (spot 12) showed higher expression in epimastigote than in trypomastigote. These results are in agreement to what is known currently about *T. cruzi *energetic metabolism. Epimastigote form which lives in the insect gut obtain their energy from aminoacids such as L-proline and/or L-glutamine, the prominent constituent of insect hemolymph and tissue fluids [29]. Consequently, epimastigote relies on amino acid catabolism. On the other hand, the trypomastigote is present in the mammal host and uses glucose, which is abundant in host fluids, as carbon source [29].

Spots 3 and 4 were identified as *T. cruzi *dehydrogenases. These proteins are NAD(P)H flavin oxidoreductases that catalyze prostaglandin PGF2α synthesis in *T. cruzi *as well as reduction of some trypanocidal drugs. It was recently shown for epimastigote that wild type *T. cruzi *displayed higher transcription level of this gene than parasite isolates with *in vitro *induced resistance to benznidazole, a drug used in the treatment of Chagas disease [30]. In our 2-DE experiments, NAD(P)H flavin oxidoreductases were present in epimastigote but not in trypomastigote, that confirms previous results using MUDPIT technology [9].

Although both epimastigote and trypomastigote are flagellate, the 69 kDa paraflagellar rod protein, a component of *T. cruzi *flagellum, was detected only in the latter (spot 30). In fact, this spot was found in the acid end of the trypomastigote 6–11 2-DE gel. It would be feasible that isoforms of this protein could be found in epimastigote but in a more acid pH range. However, pH 4–7 pH 2-DE gels of epimastigote showed the absence of matching spots (data not shown) in the corresponding region where paraflagellar proteins were detected in trypomastigote 4–7 pH 2-DE gels [3]. The stage specificity of the 69 kDa paraflagellar rod protein verified here may indicate differences in the molecular composition between both *T. cruz*i life forms.

A calpain-like protein (spot 25) was detected in epimastigote but not in trypomastigote. Calpains and calpain-like proteins comprise a large family of genes in kinetoplastids. More than 20 gene sequences were found in *T. cruzi *genome [31]. Accordingly, some members of this family were previously shown to have higher expression in epimastigote than in trypomastigote [9].

Spot 31, detected in trypomastigote but not in epimastigote 2-DE gel, is a member of the *trans*-sialidase family. The *trans*-sialidases are cell surface proteins responsible for the incorporation of sialic acid from host cells into molecules present in the parasite membrane and play a role in immune evasion and host cell entry mechanisms [32]. The previous demonstration by MUDPIT analysis that *trans-*sialidases are largely expressed in trypomastigote and absent in epimastigote [9] validates the comparative approach used here.

## Conclusion

Here we described the optimization of *T. cruzi *2-DE in the alkaline pH range. The optimized protocol basically consisted on the use of anodic "paper bridge" sample loading on Multiphor II (GE Healthcare), the use of a DTT solution embedded electrode pad near the cathode and increased concentrations of DTT and Triton X-100 in the sample buffer. The resulting epimastigote 2-DE gel permitted the visualization of approximately 700 spots in the 6–11 pH range and over 400 spots above p*I *7.0.

The "two-in-one gel" method, herein used for the comparison of epimastigote and trypomastigote 2-DE gels, minimized variations in spot migration that facilitate computational image analysis and also reduced spot volume variance that is mostly due to the 2-DE second dimension and gel staining as recently demonstrated [33].

Preliminary comparative analysis of epimastigote and trypomastigote 2-DE maps revealed differences in protein expression that are in agreement with their biological traits and also corroborated previous *T. cruzi *proteomic studies. Additional experiments using immunological approaches may be use to confirm the differential expression of the proteins identified here as well as to determine their subcellular localization.

We expect that the approach used here can be applied in future to investigate the alkaline proteome of other *T. cruzi *stages or isolates as well as in time course studies of differentiation processes such as amastigogenesis and metacyclogenesis. It is also feasible to use the protocol to the study of alkaline proteomes from other trypanosomatides such as *T. brucei *and *Leishmania spp*.

## Methods

### *In vitro T. cruzi *culture

*T. cruzi *Berenice stock epimastigotes were grown at 28°C in liver infusion tryptose (LIT) medium supplemented with 7.5% inactivated fetal bovine serum -FBS (Sorali Biotecnologia, Campo Grande, Brazil) [3]. Trypomastigotes were maintained in monolayer culture of murine L6 cells grown in DMEM medium supplemented with 7.5% (v/v) inactivated FBS, at 37°C, under 5% CO_2 _[3].

### Protein solubilization

Epimastigote and trypomastigote cells were harvested by centrifugation (3,400 × *g*, 10 min, 4°C) and washed three times in Tris-Buffered Saline (TBS, pH 7.4). For each pellet of 1 × 10^9 ^cells, 100 μL of 0.2% SDS were added. The sample was boiled for 5 min and then chilled in ice for 1 min in order to break cells and inactivate proteases [3]. A volume of 100 μL of solubilization buffer (7.77 M urea, 2.22 M thiourea, 65 mM DTT, 2% Triton X-100) supplemented with Complete Mini Protease Inhibitor cocktail (Roche, Mannheim, Germany) was added to the lysate and immediately stored at -20°C. To minimize sample variability, for each parasitic form, protein extracts from five separate growths were mixed together resulting in a pooled protein sample that was used in further 2-DE analysis. The sample volume was completed with solubilization buffer to obtain a concentration of 1.0 × 10^8 ^cells/ 100 μL. Both epimastigote and trypomastigote pooled samples were incubated under agitation for 1 h and centrifuged (12,000 × *g*, 15 min, 4°C), and the supernatants were stored at -20°C. Protein concentrations were quantified using the Plus One 2D Quant Kit (GE Healthcare, Uppsala, Sweden) according to manufacturer's instructions.

### Two dimensional gel electrophoresis (2-DE)

Several alkaline IEF conditions were tested including equipment, sample buffer, sample application, running parameters and use of electrode paper pad embedded in 2-DE sample buffer as shown in Table 1. In all methods, samples were applied to 18 cm IPG strips with a linear separation range of pH 6–11 (GE Healthcare). Prior to 2-DE, extracts containing 500 μg of protein were diluted in the appropriate final volume of 2-DE sample buffer (SB1, SB2 or SB3, see Table 1), and incubated under agitation for 1 h and centrifuged at 12,000 × *g*, 10 min at room temperature.

Following IEF, the IPG strips were equilibrated for 40 min in a reducing solution (6 M urea, 30% (v/v) glycerol, 2% (w/v) SDS and 125 mM DTT) and for additional 40 min in a alkylating solution (6 M urea, 30% (v/v) glycerol, 2% (w/v) SDS and300 mM acrylamide) [34]. Before second dimension, the IPG strips were briefly rinsed with SDS-PAGE running buffer (25 mM Tris, 192 mM glycine, 0.1% (w/v) SDS). SDS-PAGE was performed on 12% T polyacrylamide gels run on a Protean II system (Bio-Rad, Hercules, CA, USA) at 20°C. Proteins were visualized by silver staining as described elsewhere [35] and the gels were stored in 1% acetic acid before protein digestion.

To compare the epimastigote and trypomastigote alkaline 2-DE maps, "two-in-one gel" analysis was carried out as previously described [12]. Briefly, equal amounts of both samples were focused on separate IPG strips using the optimized method F (Table 1). The IPG strips were cut in the middle resulting in two halves corresponding to pH range 6–8.5 and 8.5–11 respectively. The strips bearing the same pH range were loaded side-by-side on top of a vertical SDS-PAGE gel for molecular mass separation. The "two-in-one" gels were then silver stained and stored in 1% acetic acid.

### Image analysis

Silver stained gels were scanned (PowerLook 1120, Amersham Biosciences) at 300 dpi resolution and the digitalized images were analyzed with Image Master Platinum software 5.0 (GE Healthcare) for spot detection, quantification and matching.

### Statistical analysis

Spot volume ratios between matched spots from epimastigote and trypomastigote samples (E/T) were calculated from "two-in-one" gels in triplicate. Thus, if an E/T ratio value for a given spot was equal to 1, it indicated that the protein was equally expressed in both life stages. The E/T ratio varied from 0 to 1 when trypomastigote value was higher, and between 1 to 8 when epimastigote value was higher.

Prior to one-sample t-test, Shapiro-Wilk test [28] was applied to E/T ratio to test the null hypothesis that the samples came from a normally distributed population. One-sample *t*-test was carried out using SPSS^® ^13 for Windows^® ^to determine if the mean E/T ratio of each spot was statistically different from 1 (test value = 1), or in other words, if any spot had a statistically significant differential expression (p < 0.05) between the two trypanosome forms.

### Peptide mass fingerprinting

Silver stained spots were excised, destained, in-gel digested with trypsin (Promega, Madison, USA) and extracted as previously described [3]. Briefly, the resulting peptide mixtures were analyzed in a MALDI-TOF mass spectrometer (Reflex IV, Bruker Daltonics, Karlsruhe, Germany). Each sample was concentrated and desalted with ZipTip C_18 _(Millipore, MA, USA), and eluted directly with matrix solution (20 mg/ml α-cyano-4-hydroxycinnamic acid in 0.1% TFA, 50% acetonitrile) onto the target plate prior to MALDI-TOF MS analysis as previously described [3]. Mass spectra were performed using X-TOF and Biotools software (Bruker Daltonics). Protein identification was performed using Mascot software . The searches were performed against NCBI non redundant database with 0.2 Da mass tolerance and protein mass restriction according to the observed for each spot in the gel. The search parameters were restricted to eukaryotes and allowed one missed cleavage, propionamidation of cysteine and oxidation of methionine (variable modification).

## List of abbreviations

2-DE: two-dimensional gel electrophoresis; IEF: isoelectric focusing; MS: mass spectrometry; DTT: dithiotreitol; IPG: immobilized pH gradient; TCA: trichloroacetic acid; PMF: peptide mass fingerprinting; TBS: Tris-Buffered Saline; SB: 2-DE sample buffer.

## Competing interests

The authors declare that they have no competing interests.

## Authors' contributions

ADM optimized alkaline 2-DE, image analysis, "two-in one gel" and protein identification by PMF. SC performed "two-in one gel", protein identification by PMF and participated in the writing of the manuscript. JP optimized alkaline 2-DE and participated in the writing of the manuscript. RAPG performed image analysis and protein identification by PMF. ARLT participated of *T. cruzi *cell culture and revised the final version of the manuscript. JMS participated of *T. cruzi *differentiation experiments and writing of the manuscript. MVS participated of data analysis and preparation of the manuscript. CAOR was responsible for experimental design, data analysis, coordination and preparation of the final version of the manuscript. All authors read and approved the final manuscript.

## Supplementary Material

Additional file 1 Correlation between experimentally determined and theoretical Mr (A) and p*I *(B) values of identified protein spots from *T. cruzi *2-DE gels. The correlation values were: Pearson r = 0.984, P < 0.001 for Mr and p*I *Pearson r = 0.852, P < 0.001 for p*I *values. Click here for file

Additional file 2 In order to test if the E/T values were normally distributed we applied the Shapiro-Wilk test. If p > 0.05 the hypothesis of the non-normality of the sample distribution can be rejected. Spots that are not shown in the table bellow appear only in one parasite life form. The results showed that all samples presented normal distribution and could be analyzed by the Student's t-data. Click here for file
