# Supplementary material for: Trypanosoma cruzi alkaline 2-DE: Optimization and application to comparative proteome analysis of flagellate life stages
Source: Proteome Sci. 2008 Sep 8;6:24. doi: 10.1186/1477-5956-6-24 (PMC2553069; doi:10.1186/1477-5956-6-24)
Supplement: Additional file 1 — Correlation between experimentally determined and theoretical Mr (A) and pI (B) values of identified protein spots from T. cruzi 2-DE gels. The correlation values were: Pearson r = 0.984, P < 0.001 for Mr and pI Pearson r = 0.852, P < 0.001 for pI values. [file 1477-5956-6-24-S1.doc]

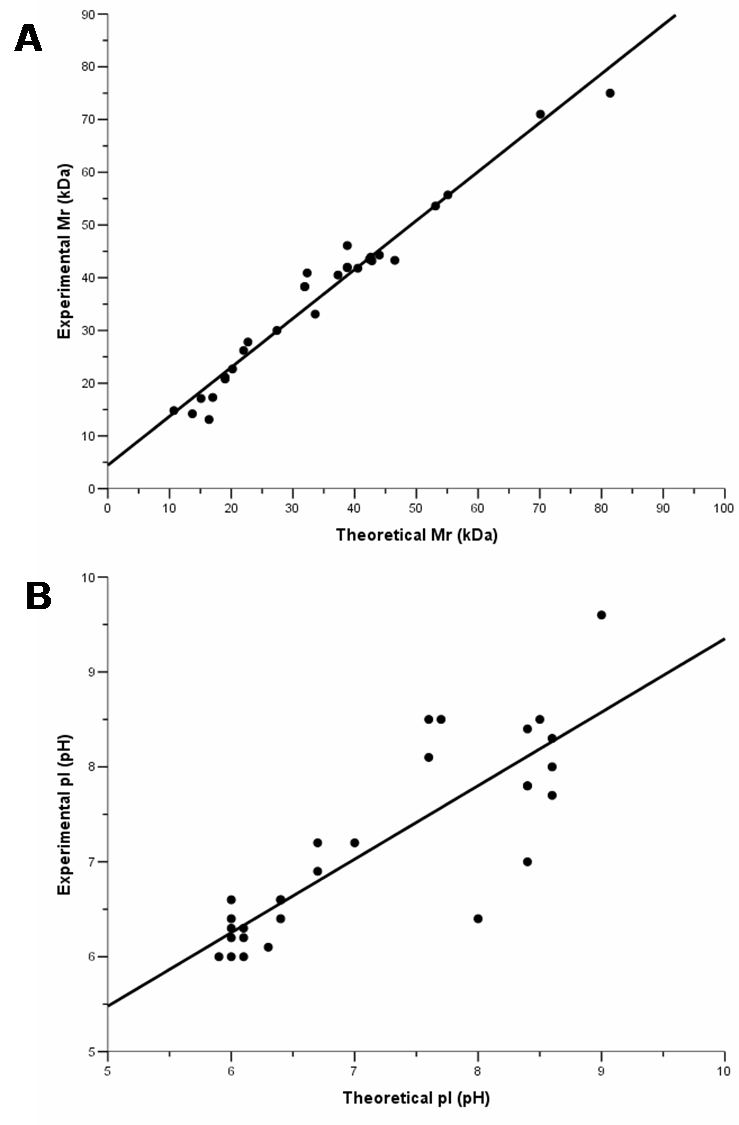


**Additional file 1 -** Correlation between experimentally determined and theoretical Mr (A) and p*I* (B) values of identified protein spots from *T. cruzi* 2-DE gels*.* The correlation values were: Pearson r = 0.984, P < 0.001 for Mr and p*I* Pearson r = 0.852, P < 0.001 for p*I* values.
