# Supplementary material for: Trypanosoma cruzi alkaline 2-DE: Optimization and application to comparative proteome analysis of flagellate life stages
Source: Proteome Sci. 2008 Sep 8;6:24. doi: 10.1186/1477-5956-6-24 (PMC2553069; doi:10.1186/1477-5956-6-24)
Supplement: Additional file 2 — In order to test if the E/T values were normally distributed we applied the Shapiro-Wilk test. If p > 0.05 the hypothesis of the non-normality of the sample distribution can be rejected. Spots that are not shown in the table bellow appear only in one parasite life form. The results showed that all samples presented normal distribution and could be analyzed by the Student's t-data. [file 1477-5956-6-24-S2.doc]

**Additional file 2 -** In order to test if the E/T values were normally distributed we applied the Shapiro-Wilk test. If p>0.05 the hypothesis of the non-normality of the sample distribution can be rejected. Spots that are not shown in the table bellow appear only in one parasite life form. The results showed that all samples presented normal distribution and could be analyzed by the Student’s t-data.

|  | | | *Shapiro-Wilk* | | |
| --- | --- | --- | --- | --- | --- |
| **SPOT** | | | *Statistic* | *df* | *Sig.(p-value)* |
|  |  | **2** | 0.926 | 3 | 0.472 |
|  |  | **5** | 0.978 | 3 | 0.714 |
|  |  | **6** | 0.957 | 3 | 0.601 |
|  |  | **7** | 0.982 | 3 | 0.746 |
|  |  | **8** | 0.941 | 3 | 0.533 |
|  |  | **9** | 0.994 | 3 | 0.856 |
|  |  | **10** | 0.804 | 3 | 0.124 |
|  |  | **11** | 0.858 | 3 | 0.263 |
|  |  | **12** | 1 | 3 | 0.987 |
|  |  | **13** | 0.907 | 3 | 0.408 |
|  |  | **14** | 0.863 | 3 | 0.276 |
|  |  | **16** | 0.955 | 3 | 0.593 |
|  |  | **17** | 0.963 | 3 | 0.63 |
|  |  | **18** | 0.975 | 3 | 0.694 |
|  |  | **19** | 0.977 | 3 | 0.71 |
|  |  | **21** | 0.943 | 3 | 0.541 |
|  |  | **22** | 0.871 | 3 | 0.299 |
|  |  | **23** | 0.848 | 3 | 0.234 |
|  |  | **24** | 0.992 | 3 | 0.824 |
|  |  | **26** | 0.951 | 3 | 0.572 |
|  |  | **29** | 0.975 | 3 | 0.694 |
